# Exploring Morpho-Physiological Variation for Heat Stress Tolerance in Tomato

**DOI:** 10.3390/plants10020347

**Published:** 2021-02-12

**Authors:** Samikshya Bhattarai, Joshua T. Harvey, Desire Djidonou, Daniel I. Leskovar

**Affiliations:** 1 Texas A&M AgriLife Research and Extension Center, Texas A&M University, Uvalde, TX 78801, USA; bsami@tamu.edu (S.B.); josh.harvey@agnet.tamu.edu (J.T.H.); 2 College of Agricultural Sciences and Natural Resources, Texas A&M University-Commerce, Commerce, TX 75428, USA; Desire.Djidonou@tamuc.edu

**Keywords:** chlorophyll fluorescence, electrolyte leakage, heat injury index, correlograms, heatmap

## Abstract

Texas tomato production is vulnerable to extreme heat in the spring-summer cropping period, which is exacerbated by the lack of superior genetic materials that can perform well in such environments. There is a dire need for selecting superior varieties that can adapt to warm environments and exhibit high yield stability under heat stress conditions. This research aimed at identifying heat-tolerant varieties under heat-stress conditions in controlled and open-field environments and was carried out in three stages. For the first experiment, 43 varieties were screened based on yield responses in natural open-field environment. From those, 18 varieties were chosen and exposed to control (greenhouse: 26/20 °C) and constant heat-stress (growth-chamber: 34/24 °C) conditions for three months. Measurements were done for chlorophyll fluorescence, chlorophyll content (SPAD), plant height, stem diameter and heat injury index (HII). The last experiment was conducted in an open field with a pool of varieties selected from the first and second experiments. Leaf gas exchange, leaf temperature, chlorophyll fluorescence, SPAD value, electrolyte leakage, heat injury index and yield were assessed. From the combined studies, we concluded that heat-tolerant genotypes selected by using chlorophyll fluorescence and HII in controlled heat-stress conditions also exhibited heat-tolerance in open-field environments. Electrolyte leakage and HII best distinguished tomato varieties in open-field environments as plants with low electrolyte leakage and HII had higher total yield. ‘Heat Master,’ ‘New Girl,’ ‘HM-1823,’ ‘Rally,’ ‘Valley Girl,’ ‘Celebrity,’ and ‘Tribeca’ were identified as high heat-tolerant varieties. Through trait correlation analysis we provide a better understanding of which traits could be useful for screening and breeding other heat-tolerant tomato varieties.

## 1. Introduction

Tomato (*Solanum lycopersicum* L.), the second-most valuable crop globally, originated in the South America’s Andean region [1]. It is a nutritious food with a multitude of uses whose demand is escalating in the world market [2]. In terms of world production, China is the leading producer of tomato, representing 31% of the total volume, followed by India (11%), the United States (9%), Turkey (7%) and Egypt (5%) [3]. The United States (USA) tomato demand is supplied through imports from other countries, with 90% of monthly imports from Mexico [4]. In the USA, tomato production is mainly concentrated in California and Florida, which constitute about two-third of the total USA tomato production [5]. Despite the large numbers of tomato growers, more than 80% of tomato demand in Texas markets is met via Mexico imports. A primary limiting factor for tomato production in Texas is erratic high-temperature extremes during the spring-summer cropping season, exacerbated by the lack of genetic materials that can perform well in such environments [6]. Thus, it is essential to identify tomato varieties that can sustain yield under high-temperature conditions.

Exposure of tomato plants to stressful temperatures triggers numerous alterations in plant physiology and morphology. Generally, tomato plants under heat-stress exhibit wilting, reduction of growth, improper development, alteration of photosynthesis and reduction in crop yield and quality [7]. However, tomato’s sensitivity to high temperature differs among genotypes [8,9,10,11], which opens the opportunity to explore, select and adopt tomato varieties with heat-tolerance in areas that experience elevated temperatures during the cropping period.

For tomato production, the optimum temperature for growth and development is between 25–30 °C during daytime and 20 °C at night [12]. An increase in temperature beyond 32 °C significantly reduces tomato fruit production [13]. At high temperatures, degradation of proteins, chlorophyll content and membrane stability and an increase in electrolyte leakage results in reduction of maximal photochemical efficiency of photosystem II (PSII) in heat-sensitive tomato plants [14]. Heat stress also affects the structural organization of the thylakoid membrane, dislodges PSII light-harvesting complexes, stimulates the synthesis of reactive oxygen species and inhibits the functionality of PSII, ultimately leading to the suppression of CO_2_ assimilation [15]. In addition to the reduced efficiency of PSII in heat-sensitive tomato plants, elevated temperature induces reproductive damage to plants including flower abortion, deformation of the anther, loss of pollen viability, low pollen germination and low fruit set [16].

Various studies have been conducted to select heat-tolerant tomato varieties, with most being executed in controlled heat stress environments like growth chambers and greenhouses [11,12]. Only a few studies have been conducted in natural heat stress conditions in open-field [8]. While screening in controlled heat stress conditions provides specific heat-related responses of plants, screening in open-field remains vital as plants are exposed to a combination of different environmental conditions along with heat. Thus, the selection of heat-tolerant varieties under controlled heat stress alone does not necessarily imply that the varieties will perform well in open-field conditions. Thus, an integrated approach using both, controlled and open-field conditions, should be followed to select the most heat-tolerant tomato varieties.

The predicted rise in the earth’s temperature, between 1.5 °C and up to 11 °C by the next century, will pose severe consequences for food production [17]. Crop yield is estimated to decrease by 17% with every one-degree increase above the optimum threshold in the growing season’s average temperature [18]. In Texas, open field tomato production is already vulnerable to extreme heat in the spring-summer cropping period, which will be exacerbated by the predicted rise in the temperature in the following three decades. Therefore, it is imperative to sustain food production in such unfavorable conditions. Thus, Texas tomato production systems should be provided with vigorous tomato cultivars that can enhance production under such unfavorable conditions, thereby meeting local demand and potentially adding to the US economy through improved export values. This study hypothesizes that there are significant differences in morpho-physiological responses among diverse tomato genotypes under high-temperature conditions in open-field and controlled environment conditions. This study aims to determine varieties that exhibit heat tolerance when grown in high-heat environments, as typically encountered in south Texas and to expand the understanding of selected traits involved in the heat-tolerance of superior varieties.

## 2. Results

### 2.1. First Open-Field Screening

**Figure 1:**
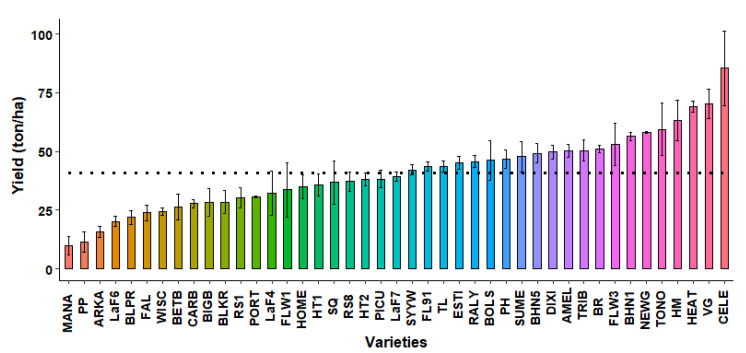
Yield (ton ha^−1^) of 43 genotypes grown in open-field during the spring season in 2019 at Uvalde, TX. Each bar represents mean ± standard error values. The dotted line indicates the total mean yield (40.71 ton ha^−1^). The varieties with similar yield responses are denoted by the same bar color.

43 different heirloom and hybrid tomato varieties were screened in an open-field in Uvalde, TX to determine their heat tolerance for fruit set and yield in a naturally heat-prone environment (Table S1). These varieties demonstrated high variability in their ability to produce high yield under the study’s environmental conditions. The average mean yield was 40.71 ton ha^−1^, which was similar to the yield of LaF7 (Figure 1). A total of 22 varieties had lower than average, ranging from 9.84 to 39.08 ton ha^−1^. MANA had the lowest yield and was the most susceptible to heat stress. In contrast, CELE had the highest yield, being 110% higher than the average. These 21 varieties seemed more promising to ensure enhanced total yields under an open-field production system, especially in regions more prone to frequent stressful temperatures, such as south Texas or other regions with comparable climates.

### 2.2. Growth Chamber and Greenhouse Screening

18 genotypes (Table S1) with high, medium and low yield were chosen from the initial field screening to study their growth and physiological heat stress responses in growth chamber environments and to contrast those with plants grown in greenhouse conditions, considered as a control. There were significant interaction effects of genotypes and temperature treatment for all the parameters assessed (Table S2).

#### 2.2.1. Chlorophyll Fluorescence (CF)

**Figure 2:**
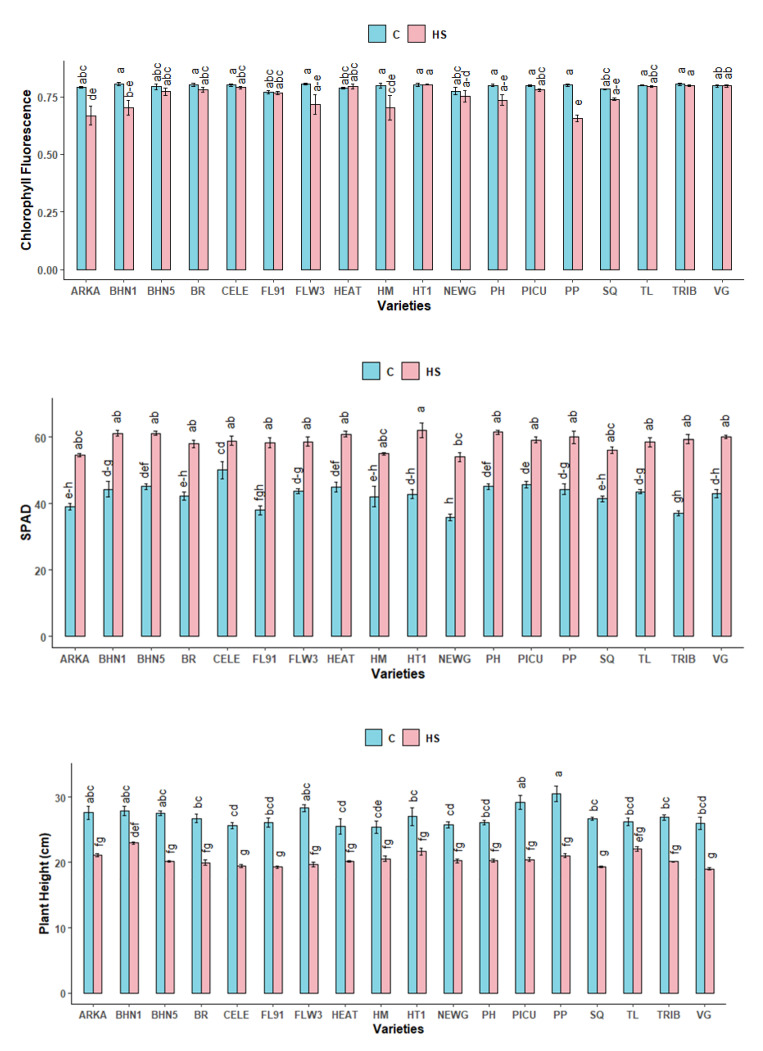

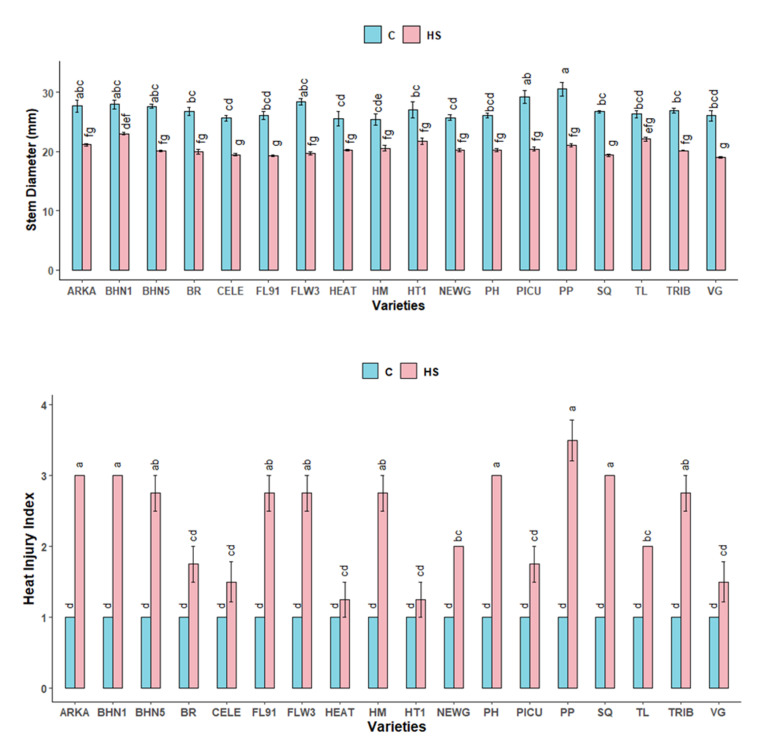
Chlorophyll fluorescence, SPAD, height (cm), stem diameter (mm) and heat injury index(HII) of different tomato varieties when exposed to two different temperature treatments: Control (C, 26/18 °C) and Heat-stress (HS, 34/24 °C). Different letters indicate significant differences between variety-temperature combinations based on Tukey’s HSD test (α ≤ 0.05). Each bar represents mean ± standard error values.

Average chlorophyll fluorescence for all varieties under the control condition was 0.795, which was 5.37% higher than that under heat-stress conditions (Figure 2). The lowest values under heat stress occurred in ARKA, BHN1, HM and PP. Chlorophyll fluorescence in these varieties were below 0.78 under heat-stress conditions, indicating reduced PSII functioning.

#### 2.2.2. SPAD

SPAD index is used as a measure of non-destructive chlorophyll content present in plant leaves. Average SPAD in all 18 varieties under heat stress was 58.77, which was 37.56% higher than the control value (Figure 2). In a multiple mean comparison between varieties, significant differences in SPAD were only observed between HT1 and NEWG under heat-stress, in which NEWG had the lower mean.

#### 2.2.3. Plant Height (Ht)

Plant height was significantly reduced under heat-stress for all varieties (Figure 2). Plant height was 24.3% higher under control compared to heat stress conditions. Reduction of plant height under heat-stress treatments was lowest in TL (15.9%) and highest in PP (31.07%).

#### 2.2.4. Stem Diameter (D)

Mean stem diameter for all varieties was 7.3% lower in heat-stress conditions (Figure 2). However, there were differences in the response of different varieties under heat stress. While almost all the varieties had lower diameter under heat-stress, SQ and NEWG had 1.14% and 28.56% higher diameter, respectively, compared to their control-treated counterparts.

#### 2.2.5. Heat Injury Index (HII)

The varieties under study varied in the severity of their macroscopic thermal injury symptoms (Figure 2). Under heat-stress, the lowest heat injury index was observed in BR, CELE, HEAT, HT1, PICU and VG, whereas the highest heat injury index was observed in PP, SQ, PH, ARKA and BHN1.

#### 2.2.6. Correlation Analysis

**Figure 3:**
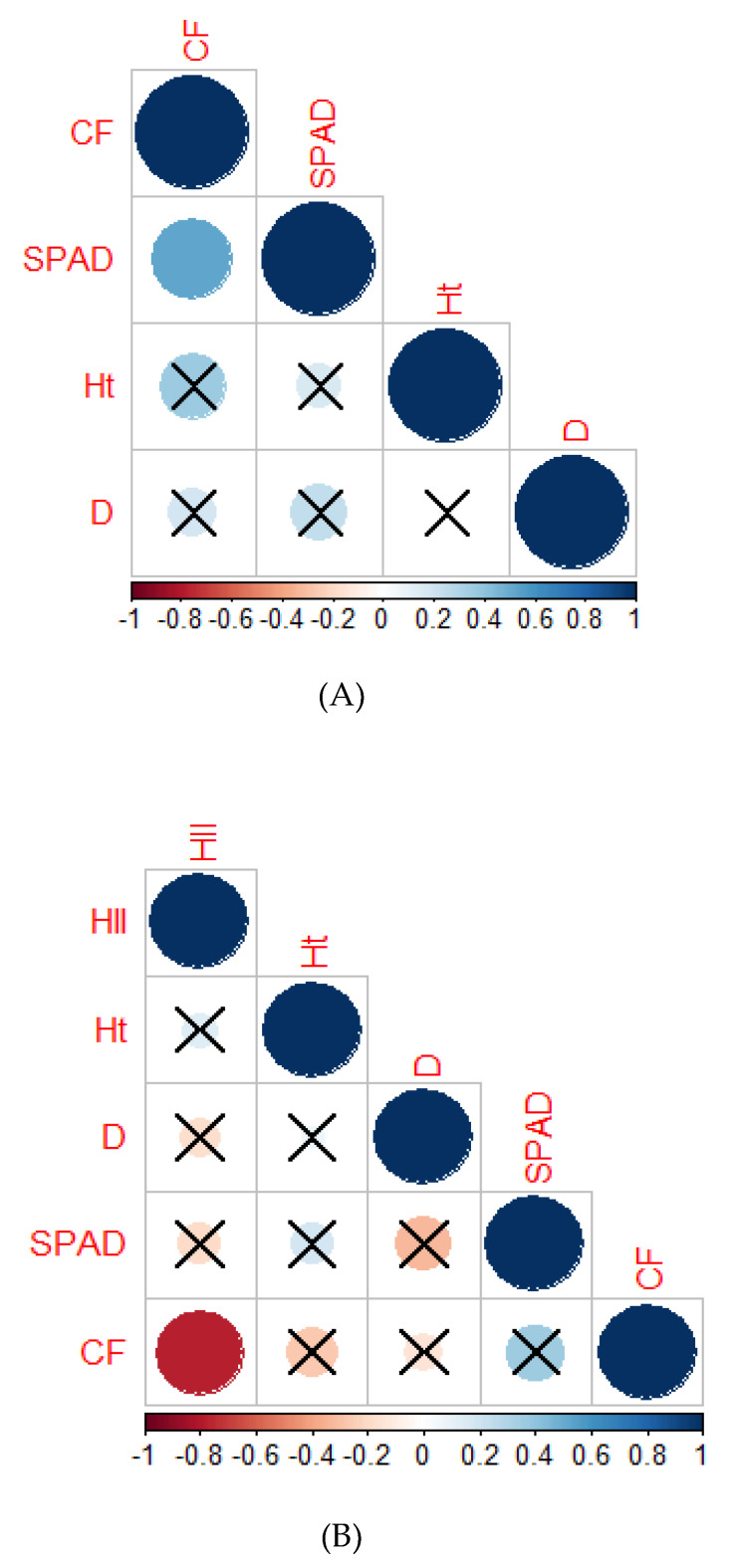
Correlograms showing the relationship between variables in the control treatment (26/18 °C, (**A**)) and heat-stress treatment (34 °C, (**B**)). The intensity of color and size of the circle increases with an increase in the significance of correlation. Dark red denotes a high negative correlation, whereas dark blue denotes a high positive correlation. The cells with cross marks denote no significant correlation between the variables. The full form of the abbreviations used in the correlograms are given in abbreviation section.

Correlograms were constructed to better understand the correlation among the variables under control and heat-stress treatments. There was a significant positive correlation (*r* = 0.55) between the SPAD and CF under control conditions (Figure 3A) but the relationship was insignificant (*r =* 0.4) under heat stress conditions (Figure 3B). A significant positive correlation was found under heat-stress conditions between CF and HII (*r* = 0.9).

### 2.3. Second Open-Field Screening

A second open-field screening was carried out in Uvalde, TX, with 24 varieties selected from the controlled environment heat stress screening and the initial open-field screening. There were significant interaction effects of variety and stage (of growth and temperature exposure) on leaf transpiration rate, maximum fluorescence, SPAD, electrolyte leakage, heat injury index and yield (Table S3). Only stage (of growth and temperature exposure) had significant effects on stomatal conductance, intercellular carbon dioxide concentration, leaf temperature, initial fluorescence, chlorophyll fluorescence and intrinsic and instantaneous water use efficiency. There was no significant effect of variety, stage or their interaction on net photosynthesis rate.

#### 2.3.1. SPAD

**Figure 4:**
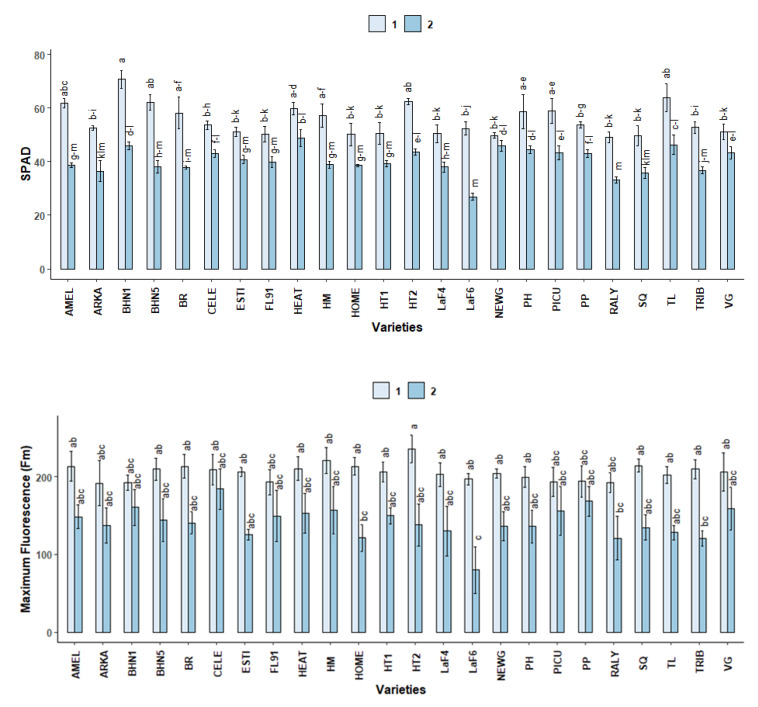
SPAD and maximum chlorophyll fluorescence (Fm) of different field grown tomato varieties at two different stages: Stage 1: 51 DAT, 34 °C and Stage 2: 86 DAT, 41°C. Different letters indicate significant differences between variety-stage combinations based on the HSD test (α ≤ 0.05). Each bar represents mean ± standard error values.

SPAD decreased significantly in the second stage in TRIB, TL, RALY, LaF6, HT2, HM, BR, BHN5, BHN1, ARKA and AMEL (Figure 4). At stage 1, the highest SPAD was observed in BHN1, followed by AMEL, BHN5, BR, HEAT, HM, HT2, PH, PICU and TL.

#### 2.3.2. Chlorophyll Fluorescence

Initial fluorescence (Fo) increased significantly for all varieties, except for CELE, HEAT, NEWG, SQ, LaF4, RALY, HOME and TL at stage 2 (Table A1). PH had the highest increase, which was 93.9% higher than stage 1. The lowest increase, 37.9%, was observed in HT1. While maximum fluorescence (Fm) decreased at stage 2 for all varieties (Figure 4), the difference was only significant in LaF6 (*p* > 0.05). All other varieties had similar Fm values at stage 1, as well as stage 2 (*p* > 0.05).

Chlorophyll fluorescence decreased significantly at stage 2 for all varieties (Table A2), with the highest decrease in LaF6 (53.4%) and the lowest in HT1 (22.2).

#### 2.3.3. Electrolyte Leakage (EL)

**Figure 5:**
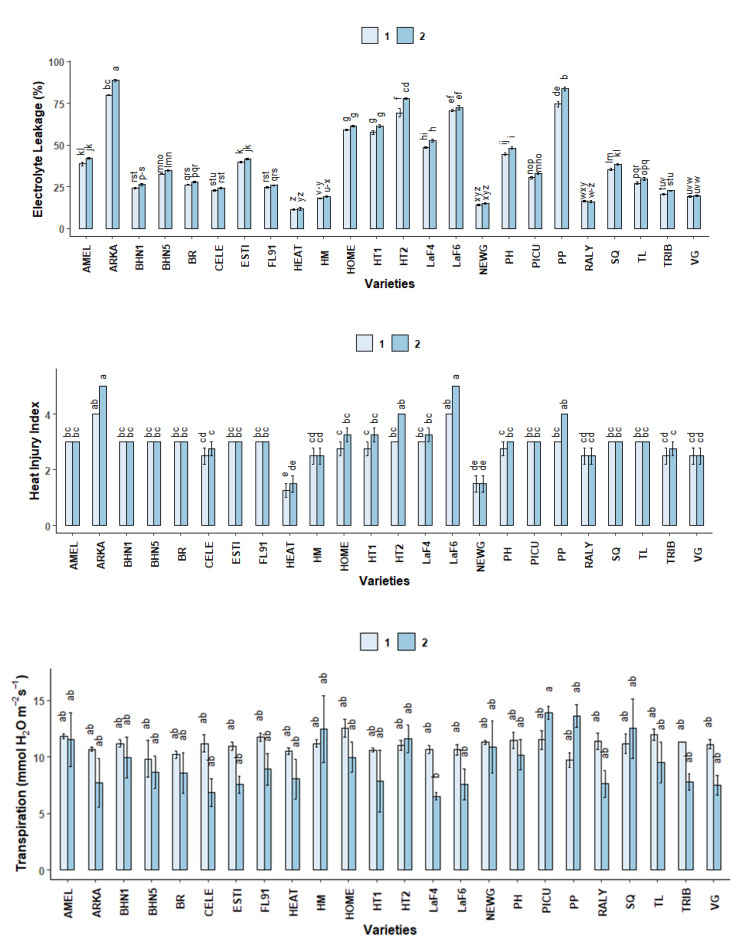
Electrolyte leakage (%), heat injury index and transpiration (mmol H_2_O m^−2^s^−1^) of different field grown tomato varieties at two different stages: Stage 1: 51 DAT, 34 °C and Stage 2: 86 DAT, 41 °C. Different letters indicate significant differences between variety-stage combinations based on the HSD test (α ≤ 0.05). Each bar represents the mean ± standard error values.

Overall, there was a subtle increase in electrolyte leakage at stage 2 but the increase was significant only for ARKA, HT2 and PP (Figure 5). ARKA had the highest electrolyte leakage among the varieties, whereas HEAT had the lowest electrolyte leakage at both stages.

#### 2.3.4. Heat Injury Index (HII)

There were no significant differences between heat injury index within varieties at the two different stages (Figure 5). At stage 1, ARKA and LaF6 had the highest heat injury index, which was statistically different from CELE, HEAT, HM, HT1, NEWG, PH, RALY, TRIB and VG. At stage 2, ARKA and LaF6 had the highest heat injury index, which was statistically similar to HT2 and PP. HEAT had the lowest heat injury index, which was statistically similar to NEWG at both stages.

#### 2.3.5. Gas Exchange

Stomatal conductance (gs) decreased abruptly at stage 2 for most varieties (Table A3). Stomatal conductance decreased most in LaF4, were it was reduced by 83.3% compared to stage 1. In contrast, PP had the lowest decrease (30.1%) in stomatal conductance.

Intercellular CO_2_ concentration (Ci) also decreased at stage 2 for LaF4, ESTI, RALY, PH, NEWG, PICU, HT2, BHN1 and AMEL (Table A4). The highest significant decrease was observed in LaF4 (74.4%), whereas the lowest decrease was observed in AMEL (31.1%).

Transpiration rate (E) differed among varieties under the two given stages (Figure 5), with some varieties exhibiting higher transpiration rates at stage 1 and others at stage 2. However, within varieties, the differences between the transpiration rates at stage 1 and stage 2 were not significantly different. The only difference was observed at stage 2 between LaF4 and PICU.

#### 2.3.6. Leaf Temperature (LT)

Leaf temperature increased significantly for all varieties at stage 2 (Table A5). The highest increase was observed in LaF4 (38.1%), whereas the lowest increase was observed in PP (22.1%).

#### 2.3.7. Water Use Efficiency

Instantaneous water use efficiency (WUEinst) did not differ significantly between and within the varieties at both stages in the mean separation test. Nevertheless, intrinsic water use efficiency (WUEintr) significantly increased in half of the varieties at stage 2 (Table A6). ARKA showed the highest increase, which was 564.7% higher than stage 1, whereas PICU showed the lowest increase, which was 181 % higher than the stage 1.

#### 2.3.8. Marketable Yield

**Figure 6:**
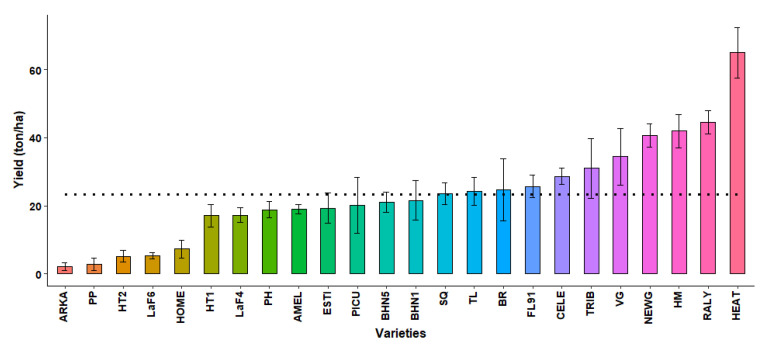
Total yield (ton ha^−1^) of 24 genotypes obtained in 2020. Each bar represents mean ± standard error values. The dotted line indicates the total mean yield (22 ton ha^−1^). The varieties with similar yield response are denoted by same bar color.

There were significant variations in total yield among varieties (Figure 6). The average yield was 23.37 ton ha^−1^. A total of 13 cultivars had yield lower than the average value, ranging from 2.6 to 21.5 ton ha^−1^. ARKA had the lowest yield and, together with PP, HT2, LaF6 and HOME, were deemed more susceptible to heat stress. In contrast, HEAT had the highest yield, followed by NEWG, RALY and HM.

#### 2.3.9. Correlation Analysis

**Figure 7:**
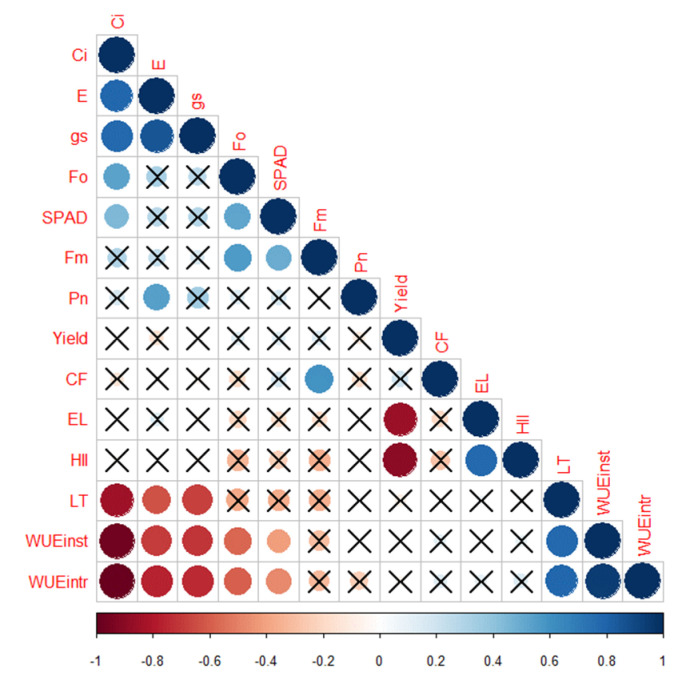
Correlogram showing the relationship between average values of the variables in open-field conditions. The intensity of color and size of the circle increases with an increase in the significance of correlation. Dark red denotes a high negative correlation, whereas dark blue denotes a high positive correlation. The cells with cross marks denote no significant correlation between the variables. The full form of abbreviations used in the correlogram are given in the abbreviations section.

A correlogram (Figure 7) depicts the correlation between all parameters measured in 24 varieties in open-field conditions. Ci was significantly and positively correlated to E (*r =* 0.75), gs (*r* = 0.8), Fo (*r* = 0.55) and SPAD (*r* = 0.5). However, there was a significant negative correlation of the parameters Ci, E and gs with LT (*r* = −0.9, −0.6 and −0.7, respectively), WUEinst (*r* = −1, −0.8 and −0.8, respectively) and WUEintr (*r* = −1, −0.8 and −0.8, respectively). The E was positively correlated to gs (*r* = 0.9) and Pn (*r* = 0.55). Additionally, there was a significant positive correlation between Fo, Fm and SPAD (*r* = 0.55 for all). CF was positively correlated with Fm (*r* = 0.55). Total yield was significantly and negatively correlated to EL (*r* = 0.9) and HII (*r* = 0.9), while these two parameters were positively correlated (*r* = 0.8). WUEintr, WUEinst and LT were positively correlated (*r* = 0.8).

## 3. Discussion

### 3.1. Growth Chamber and Greenhouse Experiment

Chlorophyll fluorescence has been widely used for heat-stress screening under controlled heat-stress conditions [8,9]. A decrease in chlorophyll fluorescence under heat stress is attributable to an increase in the initial fluorescence or a decrease in maximum fluorescence or both. It is well known that a decrease in maximum fluorescence results from an increase in non-photochemical quenching, leading to an increase in initial fluorescence due to photoinhibition of PSII [19,20,21]. Heat stress affected the PSII functionality of PP, ARKA, BHN1, HM, FLW3, PH and SQ, as shown by the lower chlorophyll fluorescence in these varieties.

Determining non-destructive chlorophyll content through SPAD measurements has been employed by many researchers based on the direct proportional relation between absolute chlorophyll content and SPAD [21]. Our results demonstrated that an increase in leaf chlorophyll content in HS conditions in all varieties may be an acclimation response of plants to high temperatures. As moderate heat stress was given, the plants could have gained an upward shift in the optimum temperature providing thermostability to PSII which most likely prevented the degradation of chlorophyll molecules [22]. Also, plant leaves under heat-stress were greener, smaller and thicker than leaves under control conditions, which may have increased chlorophyll content per unit area and, thus, SPAD value [23]. Another possible mechanism may be that the increase in temperature most likely increased the evapo-transpiration in plants in the growth chamber, which might have decreased the leaf turgor and thus interfered with the accurate chlorophyll content measurement using SPAD meter [24,25,26]. Further investigations focused on chlorophyll content changes in tomato leaves on exposure to different temperature conditions should be conducted to determine the exact cause for increase in SPAD under high temperature condition.

A reduction in plant growth under high temperatures might occur, depending on the varietal response [27]. Tomato plant height differs among different varieties. Indeterminate varieties tend to grow more than determinate plants. Our study saw differences among the varieties tested and these differences may be attributable more likely to their growth habit. However, some differences in height within the varieties under heat stress and control were significant. On average, heat stress reduced the tomato plant height [28]. Lower height reduction under heat-stress in some varieties signifies that they were able to maintain their growth properly when exposed to stressful conditions. The changes in plant diameter under heat stress may be related to changes in stem tissue hydration [29].

When plant physiology is disturbed by heat-stress, plants show visual symptoms of injury [9]. The plants with higher injury index were more sensitive to heat stress and vice-versa. In our study, HEAT and NEWG had the lowest heat injury index suggesting they are heat-tolerant varieties.

Based on correlation analysis, we found that under heat-stress plant injury increased and chlorophyll fluorescence decreased. Similarly, plants with a low heat injury index showed higher chlorophyll fluorescence. Thus, this study provides evidence that chlorophyll fluorescence could be a useful tool to assess plant sensitivity or tolerance under extended heat-stress conditions.

### 3.2. Field Experiments

**Figure 8:**
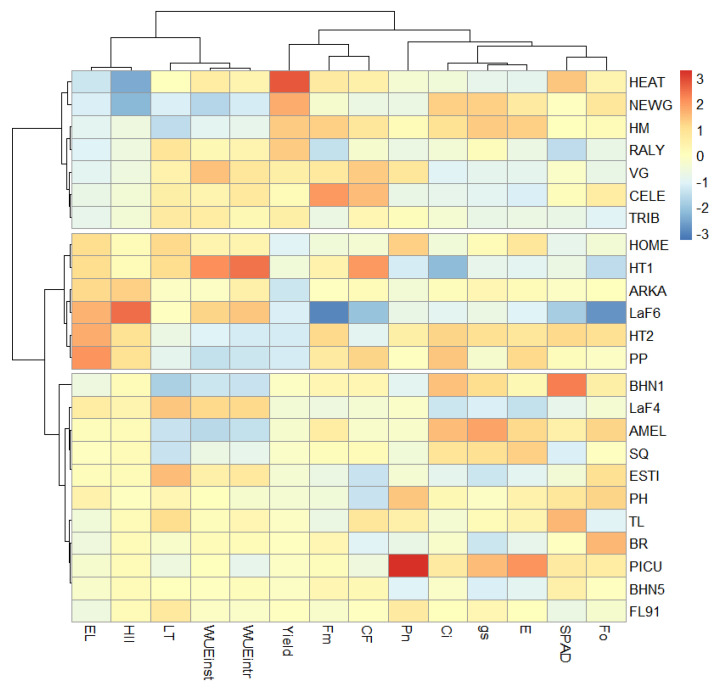
Heatmap and clustering of varieties based on the scaled values of the measured variables obtained under open-field conditions. Each row represents a variety and each column indicates a measured parameter. Treatments are clustered based on their measured variables and variables are clustered based on their correlation. The variables that are clustered together have a high positive correlation. Cells with red and blue color have high and low relative expression, respectively.

A heatmap (Figure 8) was generated to establish better relationships between the variables under different heat stresses and cluster the variables based on their responses. The heatmap distinguished three cluster groups that separated the varieties into highly heat-tolerant (first cluster from top), heat-sensitive (second cluster from top) and moderately heat-tolerant (last cluster). The clusters were clearly distinguished based on the yield, electrolyte leakage and heat injury index. The highly heat-tolerant group consisted of HEAT, NEWG, HM, RALY, VG, CELE and TRIB. Notably, these varieties were heat-tolerant under long heat-stress treatment for a more extended period in the greenhouse and growth-chamber experiment, which were mainly distinguished by chlorophyll fluorescence and HII. The highly heat-tolerant group had the lowest electrolyte leakage, lowest heat injury index and highest yield, whereas the heat-sensitive group had the highest electrolyte leakage, highest heat injury index and lowest yield.

At both stages of heat exposure, the heat-tolerant plants had an increase in transpiration as previously reported [30]. The loss of heat from the leaf surface due to enhanced transpiration led to decreased leaf temperature. An increase in transpiration likely facilitated an increase in the plants’ stomatal conductance, subsequently leading to an increase in CO_2_ diffusion into the leaves and increasing intercellular CO_2_ concentration [31]. Such an increase in intercellular CO_2_ concentration would improve the plants’ net photosynthesis rate [32]. At low leaf temperature, unaltered membrane stability in chloroplasts prevented higher electrolyte leakage [33] and thus, the PSII functionality was not affected, which was evident by higher chlorophyll fluorescence [34,35]. The lower values of electrolyte leakage and leaf temperature suggest that there was reduced chlorophyll degradation, as shown by the higher SPAD values and did not cause much injury to the plants, evidenced by the lower heat injury index. Lower injury and higher chlorophyll content further added to sustained photosynthesis in plants at a high temperature, which likely led to higher yield from the heat-tolerant plants [35].

Our study concludes that in open field screening for heat tolerance, assessing leaf electrolyte leakage with simultaneous ratings of macroscopic heat injury symptoms are the key strategies that could help select heat-tolerant varieties. However, these assessments need to be validated with total yield responses.

## 4. Materials and Methods

Three experiments were conducted to identify heat-tolerant tomato varieties after exposure to long-term heat stress conditions. The first study was conducted in 2019 in an open-field condition, followed by a second screening in a controlled heat stress environment and a final screening in open-field conditions in 2020.

### 4.1. Plant Material and Field Growing Conditions in 2019

43 different commercial and TAMU (Texas A&M University) heirloom and hybrid tomato breeding lines (Table S1) were grown in the open-field located at Texas A&M AgriLife Research and Extension Center at Uvalde, TX (29.21° N, 99.79° W) in 2019. The Uvalde climate is classified as humid subtropical in Köppen-Geiger classification [36]. The maximum, average and minimum temperatures for the whole growth period are given in Figure S1.

The seeds were sown in polystyrene 200-cell trays (2.5 × 2.5 × 7.6 cm^3^; Speedling, Ruskin, FL, USA) filled with LM-GPS (Lambert Germination, Plugs and Seedlings, Lambert, QC, Canada) media, consisting of 90% sphagnum peat moss and 10% perlite and vermiculite. The trays were saturated with water, incubated in darkness at 25 °C for two days and transferred to a greenhouse. The trays were uniformly irrigated daily with an overhead motorized spraying boom system. At the four true leaf stage, seedlings were transplanted to the field on 12 April 2019. The experiment was set up in a randomized complete block design (RCBD) with 43 varieties, three replications (blocks) and seven plants per experimental unit. Plants were spaced 0.6 m apart in rows and 1.8 m between rows. They were grown with subsurface drip irrigation (0.12 m deep) and white plastic mulch. Tomato stems were staked and strung three times (every 0.30 m). The screening was done based on the average fresh weight of fruits recorded from six harvests, performed on 27 June 2019, 2 July 2019, 11 July 2019, 17 July 2019, 25 July 2019 and 31 July 2019. Fruits from pink to the red ripe stage were picked during each harvest. Fruits were sorted into extra-large, large, medium and cull based on USDA standards [37].

### 4.2. Plant Materials and Growth Chamber and Greenhouse Conditions in 2019

After hierarchical clustering of 43 tomato varieties based on their 2019 yield, we selected 18 varieties from the resulting four clusters for further experimentation, including high-yielding (HEAT, HM, VG, CELE, BHN1 and NEWG), intermediate-yielding (BHN5, BR, TRIB, FL91, FLW3, TL and PH), low-yielding (HT1, PICU and SQ) and poor-yielding (ARKA and PP) varieties. These varieties (Figure S4) were grown in controlled environments (growth chamber and greenhouse) to compare their responses to heat stress conditions. Sowing and seedling management were done as in the first open-field experiment. Plants were transplanted to 0.8-L square pots (10.66 cm top outside, 8.68 cm bottom outside, 9.19 cm depth; TO Plastics, Clearwater, MN, USA) filled with Uvalde clay loam [38] farm soil (28% sand, 47% clay and 25% silt) on 09/18/2019 and kept in the greenhouse for five days. Half of the pots were then transferred to growth chambers (Gen1000, Conviron, Winnipeg, CA, USA) set at 26/18 °C (day/night) and allowed to acclimate for five days. The plants kept in the growth chamber were subjected to a ramping regime from 24–34 °C with 34 °C as maximum day temperature for 4 h and constant 24 °C in darkness in the growth chamber for 8 h. The pots were equally irrigated in both environments every morning to avoid desiccation. The experiment was set up in factorial CRD (Complete Randomized Design) with eighteen varieties (described in Table S1), two temperature conditions (26/18 °C in the greenhouse as control and 34/24 °C in growth-chamber as heat stress) and four replications. The growth chamber was set to a photoperiod of 16/8 h (light/dark), PAR of 350 µmol m^−1^ s^−1^ and RH of 65–75%. Data were collected for plant height, stem diameter, chlorophyll fluorescence, chlorophyll content and heat injury index starting at 30 DAT.

#### 4.2.1. Chlorophyll Content

The non-destructive chlorophyll content index was measured as an average of three leaves per plant using a SPAD meter (SPAD-502 Plus, Minolta, Japan) at 30, 60 and 95 DAT. The average of the measurements was used for analysis.

#### 4.2.2. Chlorophyll Fluorescence

Chlorophyll fluorescence (CF) was measured using the OS30p Chlorophyll Fluorometer (Opti-Sciences Inc., Hudson, NH, USA) as an average of two leaves per plant at 30, 60 and 95 DAT. The average of the measurements was used for analysis.

#### 4.2.3. Plant Height and Stem Diameter

Plant height (cm) and stem diameter (mm) was measured at the end of the experiment (95 DAT). Stem diameter was measured 2 cm above the base ground level using a caliper.

#### 4.2.4. Heat Injury Index (HII)

Plants were scored between 1 and 5 [39] at the end of the experiment, as follows:

1. = no injury
2. = yellow and mildly dehydrated margins of old leaflets
3. = mildly dehydrated plants with the middle and crinkled bottom leaflets
4. = severely dehydrated plants with upper leaflets crinkled
5. = plants with most leaves withered

### 4.3. Plant Materials and Field Growing Conditions in 2020

Twenty-four varieties were chosen from the controlled environment and open-field screening conducted in 2019. Cultural practices from sowing 15 February 2020 to transplanting 13 April 2020 in the field were done exactly like in the first open-field screening. The experiment design was set up in a randomized complete block design (RCBD) with 24 varieties, four blocks and eight plants per experimental unit. Plants were spaced 0.6 m apart in rows and 2.03 m between rows. They were grown with subsurface drip irrigation (0.12 m deep) and white plastic mulch and tomato stems were staked and strung three times (every 0.30 m). Measurements were recorded for chlorophyll content, leaf gas exchange, chlorophyll fluorescence, intrinsic and instantaneous water use efficiency, electrolyte leakage, HII (as described above) and yield. All the measurements were performed at stage 1(51 DAT, 34 °C) and stage 2 (86 DAT, 41 °C), except for yield. The plants were grown in the field until 07/28/2020. Change in daily temperature during growth period is presented in Figure S2.

#### 4.3.1. Chlorophyll Content

The non-destructive chlorophyll content index was measured as an average in two plants per variety and three leaves per plant using a SPAD meter (SPAD -502 Plus, Minolta, Japan) between 9:00–11:00 am. The average of the measurements was used for analysis.

#### 4.3.2. Leaf Gas Exchange, Chlorophyll Fluorescence and Leaf Temperature

The penultimate leaf was taken for gas exchange and chlorophyll fluorescence measurements at 56 and 86 DAT, which was performed between 10:00 am to 2:00 pm. A portable photosynthesis system (LI-6400 XT, LICOR Biosciences, NE, USA) was used to measure the net photosynthetic rate (P_N,_ µmol CO_2_ m^−2^s^−1^), stomatal conductance (gs, mol H_2_O m^−2^s^−1^), transpiration rate (E, mmol H_2_O m^−2^s^−1^) and leaf temperature (°C). Chlorophyll fluorescence (CF) was measured using the OS30p Chlorophyll Fluorometer (Opti-Sciences Inc., Hudson, NH, USA) between 9:00 am–12:00 pm.

#### 4.3.3. Intrinsic and Instantaneous Leaf Water Use Efficiency

Intrinsic leaf water use efficiency (WUEintr, µmol CO_2_ mmol H_2_O^−1^) was calculated as the ratio between P_N_ and gs and instantaneous leaf water use efficiency (WUEinst, mol CO2 mmol^−1^ H_2_O) was obtained as the ratio between P_N_ and E [40].

#### 4.3.4. Electrolyte Leakage

Electrolyte leakage (EL, %) was measured using the method of Shinohara and Leskovar (2014) [41]. Five leaf discs (4 mm) were extracted from four plants of each variety and placed in sealed culture tubes (25 ∗ 150 mm) with 10 mL of distilled water, maintained in a shaking water bath at 25 °C for 24 h and electrical conductivity (EC) of the supernatant (EC_1_) was measured. The tubes were then autoclaved at 120 °C for 20 min. The second EC (EC_2_) was measured after allowing it to cool to room temperature. The EL was determined with the equation given below:

EL (%) = (EC_1_ / EC_2_) ∗ 100.

#### 4.3.5. Average Total Yield

The average yield from a total of four harvests was recorded. Harvesting was done on 1 July 2020, 11 July 2020, 22 Janaury2020 and 28 July 2020. Fruits from pink to the red ripe stage (~35 days after anthesis) were picked during each harvest. Fruits were sorted into extra-large, large, medium and cull based on USDA standards.

### 4.4. Statistical Analysis

The data collected were analyzed in R software using one- or two-way ANOVA after verifying data for homogeneity and normality. The correlation among the variables was analyzed using Spearman correlation and a correlogram was constructed for each temperature treatment in the controlled heat-stress experiment and total plant responses from two stages for the field experiment. The multiple comparisons of means were made using Tukey’s HSD (Honestly Significant Difference) under α ≤ 0.05. For only the significant main effects of stage, mean separation for the two stages were done within each level of varieties using Tukey’s HSD (Honestly Significant Difference) at α ≤ 0.05. Clusters of varieties were obtained along with a heatmap based on the scaled value for each parameter. Correlation distance was employed in the clustering analysis.

## 5. Conclusions

Efforts to sustain crop production under steadily increasing earth’s temperature remain imperative for food security. Exploring genetic variation to determine varieties that can perform best under temperature extremes is of high priority to avoid significant food production shortages in the following years. Thus, this study was conducted to explore the potential of different tomato varieties to sustain yield under high-temperature conditions in the Texas environment, which could be considered representative of other semi-arid and warm regions of the world.

In our experiment, we obtained three significant outcomes, (a) methods to screen tomato varieties under heat-stress conditions in two contrasting environments (open-field and controlled environment), (b) determination of heat-tolerant varieties and (c) expanded understanding of which morpho-physiological traits can indicate heat-tolerance in tomato varieties in open-field conditions based on correlation analysis.

Under a controlled heat-stress environment, chlorophyll fluorescence was the most effective method to determine heat-tolerance or heat-sensitivity in the varieties. Similarly, in open-field conditions, electrolyte leakage was the best method as it is negatively correlated with total yield. Also, it is essential to note any leaf or plant injury symptoms as they are indicators of how plants respond to heat stress environments. Based on the clustering results, Heat Master, New Girl, HM-1823, Rally, Valley Girl, Celebrity and Tribeca were identified as heat tolerant varieties.

The correlation analysis provided further clues about the general mechanism of heat-tolerance in tomato. We speculate that as air temperature rises, heat-tolerant varieties have a comparatively increased transpiration rate and higher stomatal conductance than sensitive varieties which facilitates a higher diffusion of carbon dioxide into the leaves and ultimately helps to sustain photosynthesis and yield. Higher transpiration also causes loss of heat from plant leaves, reducing leaf temperature and therefore preventing the stimulation of overproduction of reactive oxygen species. Under these conditions there is no alteration of the membrane integrity. Low electrolyte leakage obtained denotes that there was no alteration in the membrane stability of PSII. Chlorophyll content is normal in stable PSII and chlorophyll fluorescence is also maintained, which depicts that the photosystem is properly functioning under high-temperature conditions in heat-tolerant varieties as compared to other heat-sensitive varieties.

From our study, we conclude that adopting varieties like Heat Master, New Girl, HM-1823, Rally, Valley Girl, Celebrity and Tribeca could increase tomato production in Texas, especially in south western areas, during the spring-summer cropping season. Further, multi-location trials with genotype by environment analysis could offer new insights into the varieties’ production potential across different locations.

## Data Availability

The data for this study are available by contacting S.B. at bsami@tamu.edu.

## Supplementary Materials

The following are available online at https://www.mdpi.com/2223-7747/10/2/347/s1, Table S1: List of varieties for the first open-field experiment, Table S2: ANOVA of different parameters measured in the second experiment (greenhouse and growth-chamber) as influenced by varieties (var) and heat-treatments (trt), Table S3: ANOVA of different parameters as influenced by varieties (var) and stages (Stage 1: 56 DAT, 34 °C and Stage 2: 86 DAT, 41 °C), Figure S1: Temperature graph for Uvalde, TX from April –July 2019, when the tomato plants were grown in the open-field for heat-stress tolerance screening. The brown, green and purple lines indicate daily maximum, average and minimum temperatures, respectively, Figure S2: Temperature graph for Uvalde, TX from April 2020–July 2020, when the tomato plants were grown in the open-field for heat-stress tolerance screening. The brown, green and purple lines indicate daily maximum, average and minimum temperatures, respectively.

## Abbreviations

CF	Chlorophyll Fluorescence
SPAD	Soil Plant Analysis Development
Ht	Plant Height
D	Stem Diameter
HII	Heat Injury Index
Ci	Intercellular CO2 Concentration
E	Transpiration Rate
gs	Stomatal Conductance
Fo	Initial Chlorophyll Fluorescence
Fm	Maximum Chlorophyll Fluorescence
Pn	Net Photosynthetic Rate
EL	Electrolyte Leakage
LT	Leaf Temperature
WUEinst	Instantaneous Water Use Efficiency
WUEintr	Intrinsic Water Use Efficiency

## Appendix A

**Table A1:** Initial/Minimum Chlorophyll Fluorescence of Different Field-Grown Tomato Varieties at Two Different Stages: Stage 1: 51 DAT, 34 °C and Stage 2: 86 DAT, 41 °C. The Values Represent the Mean ± Standard Error. Different Letters within Each Row Indicate Significant Differences between the Observed Values under Different Temperature Treatments within Each Variety. The Varieties Have Been Ordered from High to Low Percentage Increase of Initial Chlorophyll Fluorescence from Stage 1 to Stage 2.

Initial Chlorophyll Fluorescence (Fo)			
Variety	Stage 1	Stage 2	Increase (%)
PH	40.7 ± 2.4 b	79.0 ± 8.30 a	93.8
BR	42.7 ± 1.4 b	80.5 ± 9.10 a	88.3
PICU	39.7 ± 1.7 b	74.7 ± 1.40 a	88.0
BHN1	40.5 ± 1.3 b	73.7 ± 8.60 a	82.0
LaF6	41.7 ± 2.6 b	73.2 ± 7.40 a	75.4
ESTI	43.5 ± 2.0 b	74.7 ± 11.7 a	71.8
AMEL	44.5 ± 0.6 b	75.5 ± 7.50 a	69.6
ARKA	36.2 ± 4.1 b	61.5 ± 7.50 a	69.6
CELE	42.7 ± 1.8 a	72.2 ± 12.0 a	
HT2	44.0 ± 1.2 b	74.2 ± 3.50 a	68.7
FL91	40.0 ± 1.0 b	66.7 ± 5.40 a	66.8
PP	41.5 ± 0.9 b	66.7 ± 4.50 a	60.8
HEAT	41.2 ± 1.4 a	65.5 ± 10.5 a	
NEWG	45.0 ± 2.0 a	71.2 ± 12.2 a	
BHN5	42.7 ± 1.4 b	66.2 ± 7.70 a	54.9
SQ	43.5 ± 1.1 a	65.2 ± 11.2 a	
VG	41.0 ± 1.8 b	61.5 ± 3.30 a	50.0
HM	45.2 ± 1.2 b	65.7 ± 6.40 a	45.3
LaF4	43.2 ± 0.9 a	62.7 ± 11.1 a	
TRIB	41.2 ± 1.2 b	59.0 ± 4.60 a	43.0
RALY	43.0 ± 2.9 a	59.5 ± 13.5 a	
HT1	40.2 ± 2.0 b	55.5 ± 3.30 a	37.9
HOME	45.2 ± 1.8 a	60.0 ± 8.40 a	
TL	43.2 ± 1.6 a	57.0 ± 9.40 a	

**Table A2:** Chlorophyll Fluorescence of Different Field-Grown Tomato Varieties at Two Different Stages: Stage 1: 51 DAT, 34 °C and Stage 2: 86 DAT, 41 °C. The Values Represent the Mean ± Standard Error. Different Letters within Each Row Indicate Significant Differences between the Observed Values under Different Temperature Treatments within Each Variety. The Varieties Have Been Ordered from High to Low Percentage Decrease of Chlorophyll Fluorescence from Stage 1 to Stage 2.

Chlorophyll Fluorescence (CF)			
Variety	Stage 1	Stage 2	Decrease (%)
LaF6	0.79 ± 0.01 a	0.37 ± 0.10 b	53.4
PH	0.79 ± 0.01 a	0.40 ± 0.04 b	49.1
HT2	0.81 ± 0.01 a	0.42 ± 0.08 b	48.6
ESTI	0.79 ± 0.01 a	0.41 ± 0.07 b	48.4
BR	0.79 ± 0.01 a	0.43 ± 0.02 b	46.5
PICU	0.79 ± 0.01 a	0.47 ± 0.09 b	41.1
NEWG	0.78 ± 0.01 a	0.47 ± 0.06 b	39.7
HOME	0.79 ± 0.01 a	0.48 ± 0.10 b	38.9
BHN5	0.79 ± 0.02 a	0.49 ± 0.05 b	38.5
LaF4	0.78 ± 0.01 a	0.48 ± 0.06 b	38.0
AMEL	0.79 ± 0.02 a	0.49 ± 0.01 b	37.6
TRIB	0.80 ± 0.00 a	0.51 ± 0.01 b	36.5
FL91	0.79 ± 0.02 a	0.50 ± 0.07 b	36.0
SQ	0.79 ± 0.01 a	0.51 ± 0.08 b	35.9
RALY	0.77 ± 0.02 a	0.50 ± 0.06 b	35.1
HEAT	0.80 ± 0.01 a	0.53 ± 0.03 b	34.5
ARKA	0.80 ± 0.01 a	0.53 ± 0.05 b	34.0
HM	0.79 ± 0.01 a	0.56 ± 0.06 b	29.9
TL	0.78 ± 0.01 a	0.56 ± 0.05 b	28.1
BHN1	0.79 ± 0.01 a	0.58 ± 0.01 b	26.6
VG	0.79 ± 0.02 a	0.59 ± 0.04 b	25.5
PP	0.78 ± 0.02 a	0.59 ± 0.02 b	23.3
CELE	0.79 ± 0.01 a	0.61 ± 0.02 b	23.3
HT1	0.80 ± 0.01 a	0.62 ± 0.03 b	22.2

**Table A3:** Stomatal Conductance (gs) of Different Field-Grown Tomato Varieties at Two Different Stages: Stage 1: 51 DAT, 34 °C and Stage 2: 86 DAT, 41 °C. The Values Represent the Mean ± Standard Error. Different Letters within Each Row Indicate Significant Differences between the Observed Values under Different Temperature Treatments within Each Variety. The Varieties have been Ordered from High to Low Percentage Decrease of Stomatal Conductance from Stage 1 to Stage 2.

Stomatal Conductance (gs, mol H_2_O m^−2^ s^−1^)			
Variety	Stage 1	Stage 2	Decrease (%)
LaF4	0.72 ± 0.0813 a	0.1195 ± 0.0114 b	83.340
CELE	0.72 ± 0.0239 a	0.1405 ± 0.0333 b	80.372
RALY	0.79 ± 0.1043 a	0.1579 ± 0.0356 b	80.093
BHN1	0.80 ± 0.0307 a	0.1749 ± 0.0127 b	78.112
ESTI	0.67 ± 0.0361 a	0.1496 ± 0.0268 b	77.762
VG	0.70 ± 0.0402 a	0.1611 ± 0.0313 b	77.067
TRIB	0.71 ± 0.0379 a	0.1673 ± 0.0287 b	76.393
FL91	0.78 ± 0.0245 a	0.1948 ± 0.0458 b	74.910
HEAT	0.69 ± 0.0492 a	0.1756 ± 0.0565 b	74.654
LaF6	0.70 ± 0.0437 a	0.1782 ± 0.0443 b	74.599
ARKA	0.63 ± 0.0708 a	0.1820 ± 0.0670 b	70.984
HT1	0.67 ± 0.0434 a	0.1991 ± 0.1023 b	70.175
HOME	0.72 ± 0.0261 a	0.2338 ± 0.0507 b	67.725
TL	0.73 ± 0.0357 a	0.2343 ± 0.0782 b	67.719
NEWG	0.80 ± 0.0507 a	0.2588 ± 0.0657 b	67.582
PH	0.69 ± 0.0351 a	0.2478 ± 0.0611 b	63.842
BR	0.60 ± 0.0322 a	0.2216 ± 0.0712 b	62.894
BHN5	0.64 ± 0.1465 a	0.2458 ± 0.0665 b	61.858
HT2	0.74 ± 0.0342 a	0.2979 ± 0.0716 b	59.619
AMEL	0.79 ± 0.0513 a	0.3262 ± 0.1036 b	58.436
SQ	0.71 ± 0.0455 a	0.3249 ± 0.0895 b	54.073
HM	0.72 ± 0.0329 a	0.3497 ± 0.1195 b	51.246
PICU	0.71 ± 0.0370 a	0.3678 ± 0.0333 b	48.307
PP	0.54 ± 0.0437 a	0.3768 ± 0.0554 b	30.103

**Table A4:** Intercellular CO_2_ Concentration of Different Field-Grown Tomato Varieties at Two Different Stages: Stage 1: 51 DAT, 34 °C and Stage 2: 86 DAT, 41 °C. The Values Represent the Mean ± Standard error. Different Letters within each Row Indicate Significant Differences between the Observed Values under Different Temperature Treatments within Each Variety. The Varieties Have Been Ordered from High to Low Percentage Decrease of Intercellular CO_2_ Concentration from Stage 1 to Stage 2.

Intercellular CO_2_ Concentration (Ci, µmol CO_2_ mol^−1^)			
Variety	Stage 1	Stage 2	Decrease (%)
HOME	320.9 ± 3.80 a	36.30 ± 167.4 a	
HT1	320.9 ± 3.80 a	36.30 ± 167.4 a	
ARKA	318.9 ± 17.1 a	75.00 ± 121.9 a	
LaF4	323.1 ± 7.40 a	82.60 ± 69.80 b	74.4
LaF6	333.2 ± 9.70 a	93.10 ± 123.7 a	
CELE	330.3 ± 12.3 a	105.0 ± 93.40 a	
ESTI	322.0 ± 5.90 a	109.4 ± 83.40 b	66.0
VG	316.0 ± 8.50 a	109.9 ± 90.10 a	
RALY	331.7 ± 7.50 a	124.1 ± 63.80 b	62.5
TL	328.5 ± 13.2 a	131.1 ± 102.8 a	
HEAT	322.3 ± 7.75 a	132.5 ± 83.80 a	
BR	322.6 ± 10.6 a	146.6 ± 92.40 a	
TRIB	316.5 ± 5.91 a	145.4 ± 84.10 a	
FL91	322.5 ± 10.0 a	161.1 ± 67.70 a	
BHN5	330.3 ± 9.81 a	169.4 ± 80.10 a	
PH	318.9 ± 12.5 a	173.5 ± 50.20 b	45.6
SQ	330.7 ± 9.45 a	200.6 ± 80.70 a	
NEWG	340.6 ± 10.6 a	213.4 ± 22.30 b	37.3
PICU	316.7 ± 7.00 a	202.7 ± 43.30 b	35.9
HM	324.7 ± 7.70 a	208.9 ± 74.30 a	
HT2	332.9 ± 6.00 a	217.1 ± 18.30 b	34.7
BHN1	328.7 ± 8.20 a	221.1 ± 32.30 b	32.7
AMEL	336.5 ± 7.30 a	232.0 ± 29.80 b	31.1
PP	325.2 ± 10.8 a	236.4 ± 40.20 a	

**Table A5:** Leaf Temperature of Different Field-Grown Tomato Varieties at Two Different Stages: Stage 1: 51 DAT, 34 °C and Scheme 2. 86 DAT, 41 °C. The Values Represent the Mean ± Standard Error. Different Letters within Each Row Indicate Significant Differences between the Observed Values under Different Temperature Treatments within each Variety.

Leaf Temperature (°C)			
Variety	Stage 1	Stage 2	Increase (%)
LaF4	29.1 ± 0.7 b	40.2 ± 1.8 a	38.1
BR	28.8 ± 1.2 b	39.6 ± 1.6 a	37.5
CELE	28.8 ± 1.2 b	39.6 ± 1.6 a	37.5
BHN1	28.4 ± 0.6 b	39.0 ± 1.8 a	37.5
HEAT	28.6 ± 0.9 b	39.2 ± 2.1 a	36.9
RALY	29.1 ± 0.8 b	39.6 ± 2.1 a	36.0
FL91	29.2 ± 0.5 b	39.3 ± 2.2 a	34.3
ESTI	29.6 ± 0.2 b	39.7 ± 2.2 a	33.9
VG	29.1 ± 1.0 b	39.1 ± 2.2 a	33.9
HT1	29.5 ± 0.4 b	39.4 ± 1.8 a	33.2
NEWG	28.6 ± 0.8 b	38.0 ± 2.1 a	32.9
LaF6	29.1 ± 0.8 b	38.6 ± 2.3 a	32.5
TRIB	29.4 ± 0.6 b	39.0 ± 2.4 a	32.5
ARKA	29.8 ± 0.9 b	39.3 ± 2.6 a	31.6
HT2	29.0 ± 0.9 b	38.1 ± 1.7 a	31.4
BHN5	29.4 ± 1.1 b	38.2 ± 1.5 a	29.7
TL	30.0 ± 0.4 b	38.9 ± 2.4 a	29.3
PH	29.8 ± 0.9 b	38.3 ± 1.9 a	28.5
AMEL	29.1 ± 0.8 b	37.3 ± 2.5 a	28.3
HM	29.1 ± 0.9 b	37.2 ± 2.8 a	27.9
HOME	30.3 ± 0.5 b	38.6 ± 2.2 a	27.1
PICU	29.6 ± 0.2 b	37.5 ± 1.5 a	26.5
SQ	29.5 ± 0.1 b	36.9 ± 2.4 a	25.3
PP	30.1 ± 0.7 b	36.8 ± 1.9 a	22.1

**Table A6:** Intrinsic Water Use Efficiency of Different Field-Grown Tomato Varieties at Two Different Stages: Stage 1: 51 DAT, 34 °C and Stage 2: 86 DAT, 41 °C. The Values Represent the Mean ± Standard Error. Different Letters within each Row Indicate Significant Differences between the Observed Values under Different Temperature Treatments within each Variety. The Varieties Have Been Ordered from High to Low Percentage Increase of Intrinsic Water Use Efficiency from Stage 1 to Stage 2.

Intrinsic Water Use Efficiency (WUEinst, µmol CO_2_ mmol H_2_O^−1^)			
Variety	Stage 1	Stage 2	Increase (%)
LaF6	27.0 ± 4.6 a	192.2 ± 98.50 a	
HT1	33.2 ± 1.8 a	225.1 ± 113.9 a	
ARKA	34.8 ± 8.9 b	231.2 ± 123.2 a	564.6
CELE	28.2 ± 5.7 a	163.8 ± 59.01 a	
RALY	27.2 ± 4.0 b	150.5 ± 39.29 a	453.1
LaF4	32.3 ± 4.2 b	176.6 ± 43.62 a	446.8
TL	28.3 ± 6.7 a	146.0 ± 65.66 a	
ESTI	32.5 ± 2.8 a	160.3 ± 52.74 a	
VG	35.3 ± 4.0 a	159.7 ± 55.97 a	
HEAT	32.8 ± 3.9 a	145.9 ± 51.74 a	
HOME	32.7 ± 5.3 a	145.1 ± 57.50 a	
BHN5	28.1 ± 4.5 a	122.9 ± 50.79 a	
BR	32.8 ± 5.3 a	137.2 ± 59.11 a	
NEWG	22.9 ± 5.1 b	93.00 ± 13.21 a	305.1
FL91	31.4 ± 4.6 a	126.2 ± 41.32 a	
TRIB	34.8 ± 3.0 a	137.7 ± 51.55 a	
SQ	28.4 ± 4.9 a	104.7 ± 48.72 a	
PH	34.0 ± 6.0 b	118.0 ± 31.30 a	246.8
AMEL	24.5 ± 3.4 b	82.50 ± 16.93 a	235.9
HT2	26.8 ± 2.7 b	89.60 ± 10.99 a	233.8
HM	30.9 ± 3.7 a	100.2 ± 46.00 a	
BHN1	28.5 ± 3.7 b	90.60 ± 19.56 a	217.4
PICU	34.7 ± 3.8 b	97.70 ± 25.20 a	181.0
PP	31.9 ± 5.6 a	79.10 ± 24.01 a	
