# Supplementary material for: Exploring Morpho-Physiological Variation for Heat Stress Tolerance in Tomato
Source: Plants (Basel). 2021 Feb 12;10(2):347. doi: 10.3390/plants10020347 (PMC7918821; doi:10.3390/plants10020347)
Supplement: Supplementary file 1 [file plants-10-00347-s001.pdf]

Supplementary materials:

Table S1: List of varieties for the first open-field experiment.

SN	Variety	Code	Source	Growth habit	Description
**1**	Amelia*	AMEL	Seeds n Such	Determinate, 75 d	Hybrid, heat/humidity tolerant, Large, 8 to 10-oz., firm, bright
**2**	Arkansas Traveler*~	ARKA	Seeds n Such	Indeterminat e, 87 d	Hybrid, reddish-pink, smooth to a bit rough, weigh 6 to 8-oz
**3**	Better Bush	BETB	Syngenta	Determinate, 68 d	Hybrid, medium to large, 8 oz.
**4**	BHN 589*~	BHN5	BHN Seed	Semi-determinate,	Hybrid, large, 8 to 10-oz., crack-resistant fruits
**5**	BHN-1021*~	BHN1	Johnny's Seed	Determinate, 76 d	Hybrid, 8-16 oz. bright-red slicers
**6**	Big Beef	BIGB	Johnny's Seed	Indeterminat e, 73 d	Hybrid, Large, 10-12 oz. fruit
**7**	Black Krim	BLKR	Johnny's Seed	Indeterminat e, 80 d	Heirloom, Deep brown/red, 8-16 oz.
**8**	Black Prince	BLPR	Johnny's Seed	Indeterminat e, 74 d	Heirloom, 3–5 oz
**9**	Bolseno F1	BOLS	Johnny's Seed	Indeterminat e, 75 d	Hybrid, 7–12 oz. fruits
**10**	Carbon	CARB	Johnny's Seed	Indeterminat e, 76 d	Heirloom, 10-14 oz. fruit
**11**	Dixie Red	DIXI	Seeds n Such	Determinate, 70 d	Hybrid, Large, 10-oz. fruits
**12**	Estiva*	ESTI	Johnny's Seed	Indeterminat e, 70 d	Hybrid, 7-9 oz. fruits
**13**	FL 91*~	FL91	Seminis	Determinate, 72 d	Hybrid, Very large, 10-oz.
**14**	Fall 2018-253	FAL	TAMU-WES	Early Determinate	Inbreed line, large cherry type, sweet, pink fruits
**15**	TAM-FLW1	FLW1	TAMUWES	Determinate, 75 d	Hybrid, large beefsteak, red fruits
**16**	TAM-FLW3~	FLW3	TAMU-Carlos	Determinate, 75 d	Hybrid, large beefsteak, red fruits
**17**	Heat Master*~	HEAT	Seeds n Such	Determinate, 75 d	Hybrid, 7 to 8-oz., deep red fruits
**18**	HM-1823*~	HM	Clifton Seed	Determinate,	Hybrid, large to X-large
**19**	Homestead *	HOME	Seeds n Such	Determinate, 80 d	Hybrid, 8 oz., bright red fruits

Table S1 Continued

SN	Variety	Code	Source	Growth habit	Description
**20**	HT 1*~	HT1	TAMU-CS	Determinate, 75 d	Heirloom, 10 oz fruits
**21**	HT 2*	HT2	TAMU-Crosby	Determinate, 75 d	Heirloom, 8-10 oz fruits
**22**	LaF44*	LaF4	TAMU-Crosby	Determinate, 75d	Hybrid, 6 oz fruits
**23**	LaF77	LaF7	TAMU-Crosby	Determinate, 75d	Hybrid, 6-7 oz fruits
**24**	LaF66*	LaF6	TAMU-Crosby	Determinate, 75 d	Hybrid, 6-7 sized fruits
**25**	Manalucie FSt	MANA	Seeds n Such	Indeterminat e, 82 d	Hybrid, Large, bright red, globe-shaped, 1-lb. fruits
**26**	New Girl*~	NEWG	Johnny's Seed	Indeterminat e, 62 d	Hybrid, 4–6 oz. fruit
**27**	Phoenix*~	PH	Seminis Vegetable Seed	Determinate, 72 d	Hybrid, Bright red, 8-oz. fruits
**28**	Picus*~	PICU	Seeds n Such	Determinate, 79 d	Hybrid, 4 to 5-oz., deep red, roma-shaped, blocky fruits
**29**	Porter	PORT	Seeds n Such	Indeterminat e, 78 d	Hybrid, small, 2-oz., plum-shaped, smooth, dark pink
**30**	Pruden's Purple*~	PP	Johnny's Seed	Indeterminat e, 67 d	Heirloom, Large to very large (many over 1 lb.) fruits
**31**	Rally*	RALY	Sakata Seed America	Determinate, 72 d	Hybrid, 9-10 oz. large red fruits
**32**	RS 1	RS1	TAMU	Determinate , 75d	Hybrid, 5-7 oz fruits
**33**	RS 8	RS8	TAMU	Determinate , 75d	Hybrid, 6-8 oz fruits
**34**	Shourouq*~	SQ	Seminis Vegetable Seed	Determinate, 75 d	Hybrid, 8-12 oz fruits
**35**	Skyway	SYYW	Johnny's Seed	Determinate, 78 d	Hybrid, 8-12 oz. fruits
**36**	Summerpic k	SUME	Syngenta	Determinate, 75 d	Hybrid, extra-large to jumbo, 11.3 oz. fruit
**37**	Tasti-Lee*~	TL	Bejo Seeds, Inc	Determinate,	Hybrid
**38**	Tribeca*~	TRIB	Vilmorin	Determinate, 70 d	Hybrid, 9-10 oz.
**39**	Valley Girl*~	VG	Johnny's Seed	Determinate, 65 d	Hybrid, 7–8 oz. globe-shaped red fruits

Table S1 Continued

SN	Variety	Code	Source	Growth habit	Description
**40**	Wisconsin 55	WISC	Johnny's Seed	Indeterminate, 80 d	Hybrid, 6–8 oz. fruits
**41**	Bella Rosa*~	BR	Sakata Seed America, Inc.	Determinate, 74 d	Hybrid, Large, 10 to 12-oz., round, deep red, firm fruits
**42**	Celebrity*~	CELE	Clifton Seed	Determinate, 70-75 d	Hybrid, 7-8 oz, globe-shaped, firm red fruits
**43**	Tonopah	TONO	Seeds n Such	Determinate, 67 d	Hybrid, 10 oz, globe-shaped red fruits

*genotypes used in open field experiments in both 2019/2020, ~ genotypes used in control environment experiment.

Table S2: ANOVA of different parameters measured in the second experiment (greenhouse and growth-chamber) as influenced by varieties (var) and heat-treatments (trt).

Parameter	SOV	P-value	Significance
**Chlorophyll Fluorescence (CF)**	var	5.98E-06	***
	trt	1.88E-11	***
	var*trt	3.48E-06	***
**SPAD**	var	1.69E-11	***
	trt	< 2e-16	***
	var*trt	0.00428	**
**Plant Height (Ht)**	var	2.21E-08	***
	trt	< 2e-16	***
	var*trt	0.000347	***
**Stem Diameter (D)**	var	5.83E-08	***
	trt	5.86E-09	***
	var*trt	9.21E-07	***
**Heat Injury Index (HII)**	var	<2e-16	***
	trt	<2e-16	***
	var*trt	<2e-16	***

***, **,* show significant difference at P≤ 0.001, 0.01,0.05, respectively.
NS means not significant at P≤0.05.

Table S3: ANOVA of different parameters as influenced by varieties (var) and stages (Stage-1: 56 DAT, 34 °C and Stage-2: 86 DAT, 41°C).

SN	Parameter	SOV	P-value	Significance
**1.**	Net Photosynthesis Rate (Pn)	Var	0.8253	NS
		Stage	0.0605	NS
		Block	<2e-16	***
		Var × Stage	0.804	NS
**2.**	Stomatal Conductance (gs)	Var	0.1892	NS
		Stage	<2e-16	***
		Block	0.0786	NS
		Var × Stage	0.0833	NS
**3.**	Intercellular CO_2 Concentration (Ci)	Var	0.771	NS
		Stage	<2e-16	***
		Block	1.31E-13	***
		Var × Stage	0.892	NS
**4.**	Transpiration rate (E)	Var	0.00752	**
		Stage	5.88E-06	***
		Block	2.76E-05	***
		Var × Stage	0.02974	*
**5.**	Leaf Temperature (LT)	Var	1	NS
		Stage	< 2e-16	***
		Block	3.36E-09	***
		Var × Stage	0.999	NS
**6.**	Initial Fluorescence (Fo)	Var	0.0977	NS
		Stage	<2e-16	***
		Block	2.46E-09	***
		Var × Stage	0.1675	NS
**7.**	Maximum Fluorescence (Fm)	Var	0.00143	***
		Stage	< 2e-16	***
		Block	< 2e-16	***
		Var × Stage	0.00337	**
**8.**	SPAD value	Var	7.31E-11	***
		Stage	< 2e-16	***
		Block	0.58358	NS
		Var × Stage	0.00545	**

Table S3: Continued

SN	Parameter	SOV	P-value	Significance
**9.**	Chlorophyll Fluorescence (CF)	Var	0.131	NS
		Stage	< 2e-16	***
		Block	7.02E-07	***
		Var × Stage	0.134	NS
**10.**	Instantaneous Water Use Efficiency (WUE_{inst})	Var	0.836	NS
		Stage	8.30E-08	***
		Block	< 2e-16	***
		Var × Stage	0.97	NS
**11.**	Intrinsic Water Use Efficiency WUE_{intr}	Var	0.682	NS
		Stage	< 2e-16	***
		Block	6.15E-12	***
		Var × Stage	0.796	NS
**12.**	Electrolyte Leakage (EL)	Var	< 2e-16	***
		Stage	< 2e-16	***
		Block	0.2	NS
		Var × Stage	7.68E-13	***
**13.**	Heat Injury Index (HII)	Var	< 2e-16	***
		Stage	1.11E-06	***
		Block	0.92813	NS
		Var × Stage	0.00262	**
**14.**	Marketable Yield	Var	3.4e-12	***
		Block	0.124	

***, **,* show significant difference at P≤ 0.001, 0.01,0.05, respectively.
NS means not significant at P≤0.05.

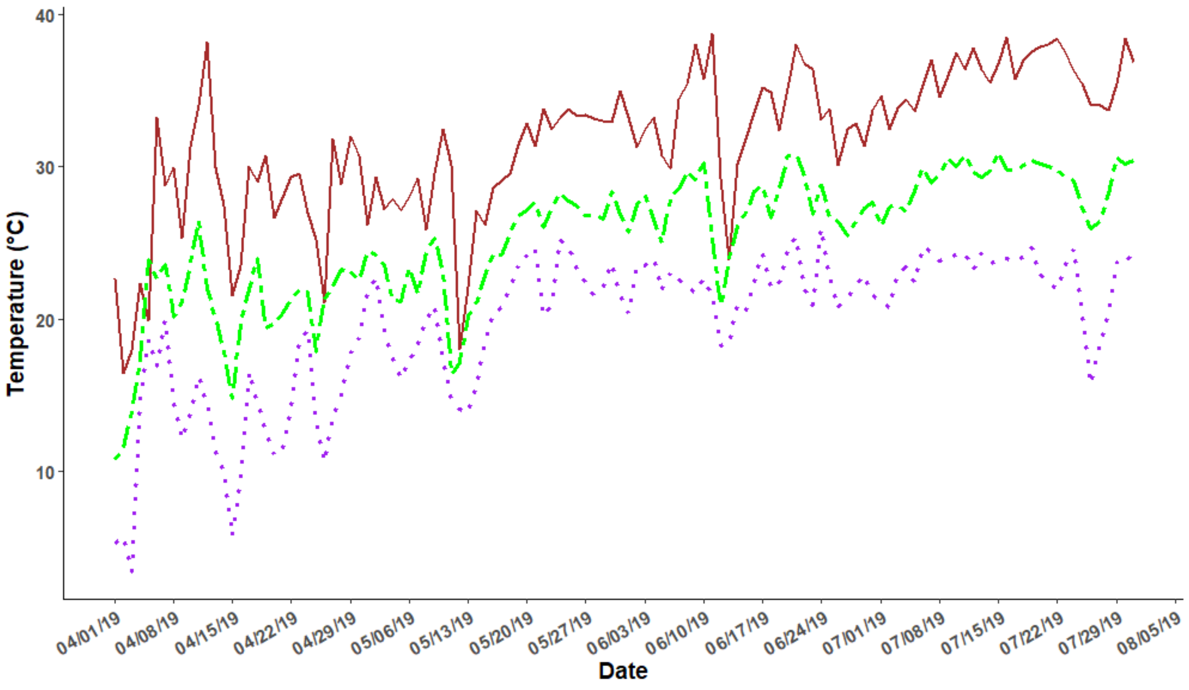

Figure S1: Temperature graph for Uvalde, TX from April 2019–July 2019, when the tomato plants were grown in the open-field for heat-stress tolerance screening. The brown, green, and purple lines indicate daily maximum, average, and minimum temperatures, respectively.

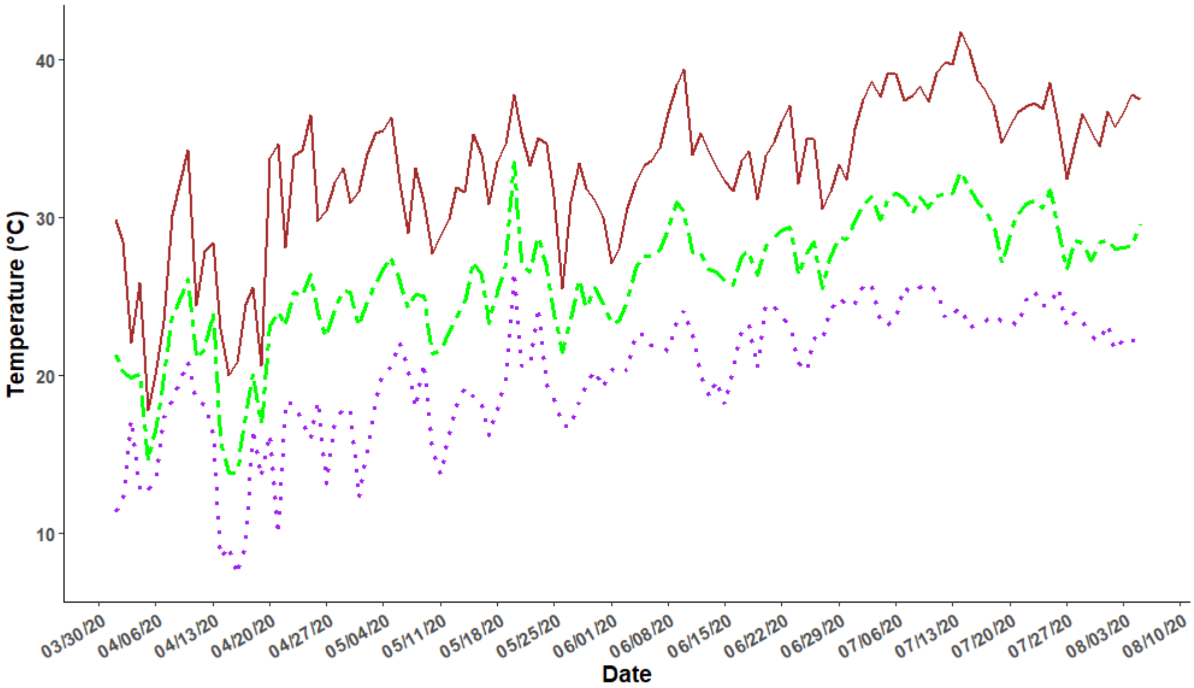

Figure S2: Temperature graph for Uvalde, TX from April 2020–July 2020, when the tomato plants were grown in the open-field for heat-stress tolerance screening. The brown, green, and purple lines indicate daily maximum, average, and minimum temperatures, respectively.
